# NADPH oxidase gp91^phox^ contributes to RANKL-induced osteoclast differentiation by upregulating NFATc1

**DOI:** 10.1038/srep38014

**Published:** 2016-11-29

**Authors:** In Soon Kang, Chaekyun Kim

**Affiliations:** 1 Laboratory of Leukocyte Signaling Research, Department of Pharmacology, Inha University School of Medicine, Incheon 22212, Korea; 2 Convergent Research Center for Metabolism and Immunoregulation, Inha University, Incheon 22212, Korea

## Abstract

Bone-marrow derived monocyte-macrophages (BMMs) differentiate into osteoclasts by M-CSF along subsequent RANKL stimulation possibly in collaboration with many other unknown cytokines released by pre- or mature osteoblasts. The differentiation process requires receptor activator of nuclear factor kappa-B ligand (RANKL)/RANK signaling and reactive oxygen species (ROS) such as superoxide anion (O_2_^•−^). Gp91^phox^, a plasma membrane subunit of NADPH oxidase (Nox), is constitutively expressed in BMMs and plays a major role in superoxide anion production. In this study, we found that mice deficient in gp91^phox^ (*gp91*^*phox*−/−^) showed defects in osteoclast differentiation. Femurs of these mice produced osteoclasts at about 70% of the levels seen in femurs from wild-type mice, and accordingly exhibited excessive bone density. This abnormal bone growth in the femurs of *gp91*^*phox*−/−^ mice resulted from impaired osteoclast differentiation. In addition, *gp91*^*phox*−/−^ mice were defective for RANKL-induced expression of nuclear factor of activated T cells c1 (NFATc1). However, H_2_O_2_ treatment compensated for gp91^phox^ deficiency in BMMs, almost completely rescuing osteoclast differentiation. Treating wild-type BMMs with antioxidants and superoxide inhibitors resulted in a differentiation defect resembling the phenotype of *gp91*^*phox*−/−^ BMMs. Therefore, our results demonstrate that gp91^phox^-derived superoxide is important for promoting efficient osteoclast differentiation by inducing NFATc1 as a downstream signaling mediator of RANK.

Osteoclasts are multinucleated bone cells that originate from bone marrow-derived monocyte-macrophages (BMMs). Osteoclast differentiation from hematopoietic progenitor cells requires macrophage colony stimulating factor (M-CSF) and receptor activator of nuclear factor kappa-B ligand (RANKL)^1^,^2^. M-CSF induces the formation of BMMs and the expression of RANK in early-stage osteoclast precursor cells^3^. M-CSF activates osteoclast precursors during early phases of osteoclast differentiation. RANKL induces differentiation of osteoclast precursors into osteoclasts and suppresses osteoclast apoptosis through NF-κB and NFATc1 signaling^4^. Osteoclasts break down bone tissue by secreting proteases, a process known as bone resorption. This function is critical for normal bone repairing and remodeling. Cathepsin K (CTSK) is the major protease involved in degrading type I collagen and other noncollagenous proteins. Knockout of CTSK in mice leads to an osteopetrotic phenotype, which is partially compensated for by increased expression of other bone proteases. Bone resorption is a multistep process associated with many enzymatic activities including proteolytic degradation by CTSK. NFATc1-mediated CTSK gene expression is induced by RANKL stimulation^5^. CTSK is secreted by osteoclasts to degrade collagen and other matrix proteins during bone resorption. Therefore, bone density and especially bone resorption are tightly regulated by the RANK–RANKL signaling pathway.

In the last decade, studies have indicated that reactive oxygen species (ROS), including superoxide and hydrogen peroxide, are crucial components that regulate the differentiation process of osteoclasts (see^6^ for a review). Reactive oxygen species (ROS) mediate RANK–RANKL-induced signaling pathways and cellular events as a second messenger in osteoclast precursors^7^,^8^. Conversely, RANKL stimulation transiently increases intracellular ROS levels through NADPH oxidase (Nox), and ROS act as second messengers in RANKL-induced signaling pathways in osteoclast precursors, regulating their consequent differentiation into osteoclasts^7^,^8^,^9^,^10^. Similarly, M-CSF also transiently increases intracellular ROS levels through Nox^3^. These findings indicate that Nox-derived ROS are essential intracellular mediators throughout the osteoclast differentiation process.

Nox consists of integral membrane enzymes that produce superoxide (O_2_^•−^) by transporting electrons to oxygen from NADPH across the membrane. Nox1-5 have been described in mammals^11^. Among those, Nox1, Nox2, and Nox4 have been found in osteoclast precursors and osteoblasts. Gp91^phox^, also known as Nox2, is highly expressed in neutrophils and macrophages, where it generates superoxide that is essential for killing pathogens by the respiratory burst^12^. Upon cellular activation, three cytoplasmic subunits, p47^phox^, p67^phox^, and p40^phox^, translocate to the membrane and join together with membrane-bound flavocytochrome *b*, p91^phox^, and p22^phox^ to form active Nox2^13^.

Recently, it has been reported that cellular Nox2 levels are regulated by a protein called negative regulator of ROS (NRROS)^14^. NRROS is located in the endoplasmic reticulum, where it negatively regulates ROS generation by degrading Nox2 during the inflammatory response. More recently, Kim *et al*. reported that NRROS is increased during osteoclast differentiation and represses Nox1 and Nox2 expression, thereby attenuating RANKL-induced osteoclast differentiation^15^. These findings suggest that Nox2 expression and Nox2-derived ROS are tightly controlled by negative regulatory networks. Mitochondria are also known to regulate Nox2 activation. Mitochondrial ROS induce slower but long-lasting Nox2 activity, suggesting cross-talk between mitochondria and Nox2^16^. More research is required in order to understand the precise regulatory networks that control Nox2 activity during osteoclast formation.

RANK is a TNF receptor family member that recognizes RANKL through cell-to-cell interaction with stromal cells, and promotes the differentiation of osteoclast precursors into osteoclasts in the presence of M-CSF^17^. RANK expression is induced in osteoclast precursors by M-CSF^3^. RANKL stimulation recruits adaptor molecules such as TNF receptor-associated factor 6 (TRAF6), and activates downstream signaling cascades including MAPKs, PI3K, and NF-κB^18^. These intracellular signaling pathways consequently upregulate osteoclastogenic transcription factors, including NFATc1^19^,^20^. NFATc1 expression in RANKL-stimulated osteoclast precursors is crucial for osteoclast differentiation, as NFATc1 induces the expression of osteoclast-specific genes such as tartrate-resistant acid phosphatase (TRAP), CTSK^17^,^21^,^22^ and osteoclast-associated receptor (OSCAR)^23^,^24^. NFATc1-deficient embryonic stem cells fail to differentiate into osteoclasts in response to RANKL stimulation. Ectopic expression of NFATc1 allows osteoclast precursors to undergo efficient differentiation in the absence of RANKL signaling. Thus, NFATc1 may represent the master switch that functions downstream of RANKL to regulate osteoclast differentiation.

Fusion of osteoclast precursors is a critical cellular event for osteoclast differentiation, and understanding its regulation will have an important impact on the development of a new therapy to control bone loss. Osteoclast fusion is regulated by various factors. Dendritic cell–specific transmembrane protein (DC-STAMP), a putative seven-transmembrane protein has been identified as an essential factor for osteoclast fusion in bone homeostasis and RANKL is a strong inducer of DC-STAMP production^25^,^26^. Although osteoclast differentiation is known to require RANKL and M-CSF, non-canonical pathways of osteoclast formation were reported in which cytokines/growth factors can substitute for RANKL to induce osteoclast formation^27^. Substitutes for RANKL include LIGHT, TNF-α and interleukins 6, 11 and 8^28^. Recently, RANKL has been reported that it can even suppress osteoclast differentiation by binding leucine-rich repeat-containing G-protein-coupled receptor 4 (LGR4, also called GPR48), another receptor for RANKL to compete RANK. RANKL binding to LGR4 activates the Gαq and GSK3-β signaling pathway, an action that suppresses the expression and activity of NFATc1 during osteoclastogenesis^29^.

In this study, we demonstrate that gp91^phox^ is involved in osteoclast differentiation by enhancing RANKL-induced NFATc1 expression in osteoclast precursors. This suggests that gp91^phox^-derived superoxide is crucial for osteoclast precursors to differentiate into osteoclasts in collaboration with RANKL/ NFATc1 signal pathways.

## Results

### Superoxide production was impaired in *gp91^phox−/−^* osteoclasts

**Figure 1:**
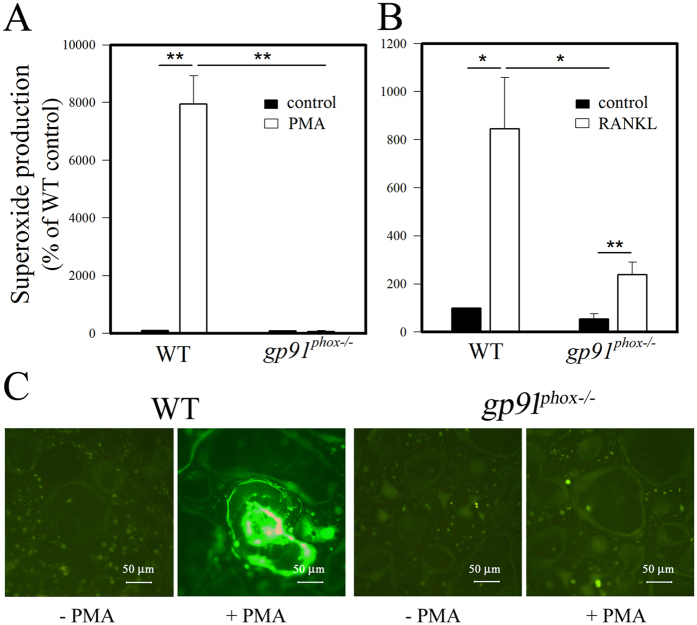
Impaired RANKL-induced ROS production in osteoclast precursors and osteoclasts from *gp91*^*phox*−/−^ mice. Osteoclast precursors were incubated with (**A**) PMA or (**B**) RANKL after preloading with isoluminol, and superoxide production was measured. Result represent the mean ± SD of 6 (**A**) and 3 (**B**) independent experiments, *p < 0.05 and **p < 0.01 (paired *t*-test). (**C**) Osteoclasts obtained from wild-type and *gp91*^*phox*−/−^ mice were pretreated with DCF-DA and observed under fluorescence microscope at the magnitudes designated after PMA treatment for 10 min. The fluorescent images are representative of least 3 independent experiments, each performed in triplicate.

Although ROS are clearly linked to osteoclast differentiation, we wanted to test if gp91^phox^-derived superoxide participates in osteoclast differentiation and bone resorption. Thus, we compared superoxide production between osteoclast precursors derived from *gp91*^*phox*−/−^ mice and osteoclast precursors derived from wild-type mice after stimulation with 200 ng/mL phorbol 12-myristate 13-acetate (PMA) (Fig. 1A) or 50 ng/mL RANKL (Fig. 1B) for 10 min and 30 min, respectively, by isoluminol chemiluminescence assay. In contrast to wild-type cells, gp91^phox^-deficient osteoclast precursors showed decreased superoxide production in response to PMA or RANKL. We stimulated osteoclast precursors with M-CSF plus RANKL for an additional 4 days to produce osteoclasts and examined intracellular ROS levels in fully differentiated osteoclasts from *gp91*^*phox*−/−^ mice and wild-type mice after PMA stimulation. As shown by fluorescence microscopic images visualized with DCF-DA (Fig. 1C), osteoclasts derived from *gp91*^*phox*−/−^ mice did not produce ROS in response to PMA as readily as osteoclasts from wild-type mice. These results indicate that gp91^phox^ is an essential Nox homologue for ROS production in both osteoclast precursors and osteoclasts in response to RANKL and PMA stimulation.

### *Gp91^phox−/−^* mice defective osteoclast formation had elevated bone volume

**Figure 2:**
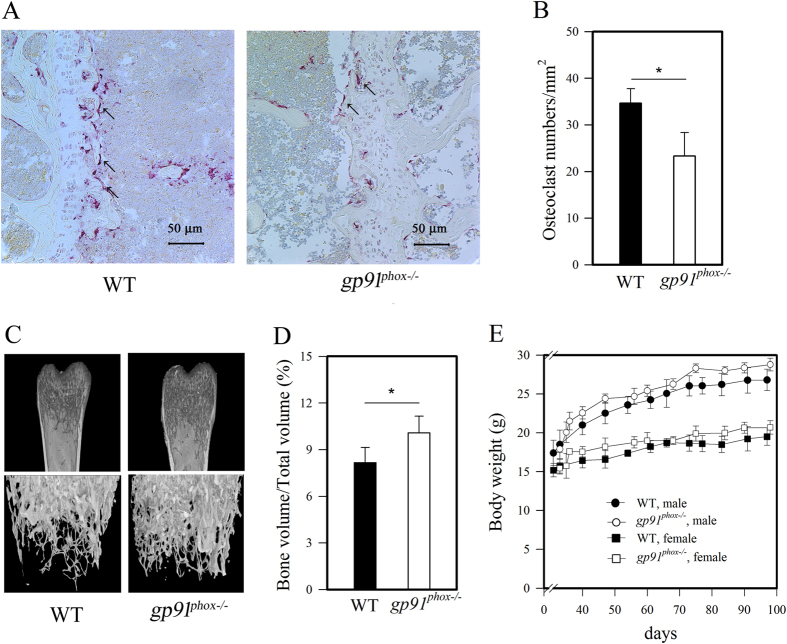
Osteopetrotic phenotypes of *gp91*^*phox*−/−^ mice. (**A~D**) Data are expressed as the mean ± SD, *p < 0.05 (paired *t*-test). (**A**) TRAP expression in femurs from 6 mice (24-week-old, wild-type male = 3; *gp91*^*phox*−/−^ male = 3, n = 3 independent replicates) detected from histological sections. (**B**) Osteoclasts from femurs per selected field were counted (n = 3 independent replicates). (**C**) Microcomputed tomography of femurs from wild-type and *gp91*^*phox*−/−^ mice was assessed (6-week-old, wild-type male = 4; *gp91*^*phox*−/−^ male = 4, n = 4 independent replicates). (**D**) Trabecular bone volumes of wild-type (male = 4) and *gp91*^*phox*−/−^ (male = 4) mice were compared by quantitative μCT as % of bone volume fraction (BV/TV). (**E**) Body weights of 42 wild-type (male = 19, female = 23) and 41 *gp91*^*phox*−/−^ mice (male = 22, female = 19) were compared for 100 days after birth. Data were analyzed by one-way ANOVA and expressed as the mean ± SD.

Since BMMs from *gp91*^*phox*−/−^ mice showed defects in ROS production, we tested the ability of BMMs from *gp91*^*phox*−/−^ mice to differentiate into osteoclasts. To accomplish this, we compared osteoclast populations and bone density between femurs from *gp91*^*phox*−/−^ mice and femurs from wild-type mice. We detected osteoclasts in the femurs by staining TRAP, an osteoclast marker (Fig. 2A) and then counted TRAP-positive cells in the histological sections (Fig. 2B). We found that *gp91*^*phox*−/−^ femurs displayed only about 68% of the number of osteoclasts seen in wild-type femurs (34.7 ± 3.1 for wild-type vs. 23.3 ± 5.0 for *gp91*^*phox*−/−^ femurs). As superoxide is crucial for osteoclast differentiation^3^,^9^,^10^,^12^,^30^,^31^,^32^,^33^, the lower number of osteoclast in *gp91*^*phox*−/−^ femurs might be due to a lack of gp91^phox^*-*derived superoxide. Osteoclasts degrade bone, a phenomenon referred to as bone resorption^34^,^35^. Thus, we tested if the decreased number of osteoclasts in the femurs of *gp91*^*phox*−/−^ mice corresponded to an increase in bone mass. Radiographic μCT analyses indicated that femurs from *gp91*^*phox*−/−^ mice showed greater bone density than femurs from wild-type mice (Fig. 2C). These data were plotted as % of bone volume fraction (BV/TV); trabecular bone volume (BV) to total volume (TV) ratio. The femurs from *gp91*^*phox*−/−^ appeared higher in bone density by 24% than those from wild-type femurs (Fig. 2D). Although we did not definitely verify causative links between the impaired superoxide and osteoclast productions and the higher bone density in *gp91*^*phox*−/−^ femurs, these concurrent events strongly suggest that higher bone density developed by *gp91*^*phox*−/−^ mice compared to wild-type mice was due to their defective osteoclast differentiation by BMMs. There were no significant differences in body weight between *gp91*^*phox*−/−^ mice and wild-type mice (Fig. 2E). Thus, the differences in bone density observed between *gp91*^*phox*−/−^ femurs and wild-type mice were not due to differences in total body weight.

### Gp91^phox^ may play an important role for osteoclast precursors to differentiate into osteoclasts

**Figure 3:**
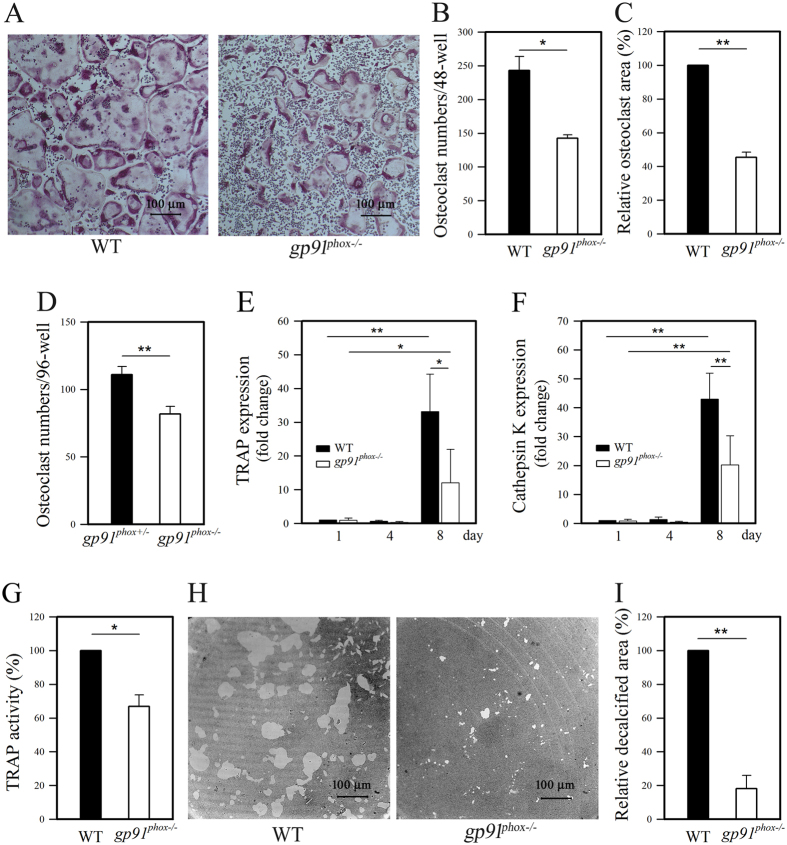
Impaired RANKL-induced osteoclast differentiation in *gp91*^*phox*−/−^ mice. Data are expressed as the mean ± SD, *p < 0.05 and **p < 0.01 (paired *t*-test). (**A–C**) Degree of RANKL/MCSF-induced osteoclast differentiation from wild-type and *gp91*^*phox*−/−^ BMMs was assessed on 8th day following RANKL stimulation by histological examination (A), cell numbers per well (**B**), and relative osteoclast areas (**C**). Result represent the mean ± SD of 3 independent experiments, each performed in triplicate. (**D**) Osteoclast numbers from 8 littermates (6-week-old, *gp91*^*phox*+/−^ female = 4; *gp91*^*phox*−/−^ male = 4) were determined (n = 4 independent replicates). Expression of TRAP (**E**) and CTSK (**F**) were measured by qPCR during osteoclast differentiation (n = 5 independent experiments, each performed in duplicate). (**G**) TRAP activity in osteoclasts was determined by measuring acid phosphatase activity (n = 3 independent experiments, each performed in triplicate). (**H**) BMMs from wild-type and *gp91*^*phox*−/−^ were differentiated into osteoclasts. Resulting cells were assessed for resorption activity (n = 3 independent experiments, each performed in triplicate). (**I**) Relative resorption areas were calculated and plotted as % of wild-type control (n = 3 independent experiments, each performed in triplicate).

Osteoclasts contain vacuoles filled with TRAP and CTSK. We utilized this to assess osteoclast differentiation by staining for TRAP as mature osteoclast marker. We counted the number of TRAP-positive multinucleated cells (more than three nuclei) which represent mature osteoclasts. Histological analysis revealed that BMMs from *gp91*^*phox*−/−^ mice displayed a significantly decreased number of TRAP-positive osteoclasts after stimulation with RANKL (~60% compared to wild-type cells) (Fig. 3A,B). Additionally, TRAP-positive osteoclasts from *gp91*^*phox*−/−^ mice were significantly smaller than osteoclasts from wild-type cells (Fig. 3A,C). We compared heterozygous *gp91*^*phox*+/−^ mice with littermate *gp91*^*phox*−/−^ mice for the osteoclast numbers, TRAP and CTSK expression after RANKL stimulation. Heterozygous *gp91*^*phox*+/−^ mice displayed significantly more osteoclasts (Fig. 3D~136% compared to knockout mice), expression levels of TRAP (Fig. 3E)and CTSK (Fig. 3F) compared to littermate *gp91*^*phox*−/−^ mice.

Consistent with the decreased number of TRAP-positive cells, *gp91*^*phox*−/−^ osteoclasts contained lower TRAP activity (~67% of wild-type) after stimulation with RANKL than wild-type osteoclasts (Fig. 3G). TRAP activity is abundant in osteoclasts. Since we calculated TRAP activity per cell, the data suggest that a large portion of BMMs from *gp91*^*phox*−/−^ mice might remain undifferentiated even in the presence of M-CSF and RANKL. We do not rule out the possibility that osteoclasts derived from of *gp91*^*phox*−/−^ mice were much smaller with less TRAP than those from wild-type mice. Femurs from *gp91*^*phox*−/−^ mice showed less resorptive area than femurs from wild-type mice as observed by histochemical images (Fig. 3H,I). Taken together, these results suggest that gp91^phox^ may play an important role during the differentiation of osteoclast precursors into osteoclasts.

### ROS are indispensable for the differentiation of BMMs into osteoclasts

**Figure 4:**
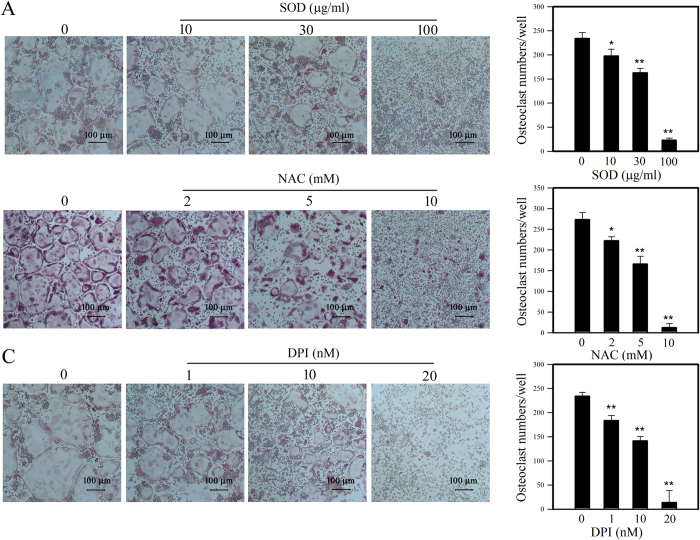
Effect of antioxidants and ROS inhibitors on osteoclast differentiation. BMMs were treated with SOD, NAC, and DPI at different concentrations and then osteoclast differentiation was induced. Resulting cells were visually examined for osteoclast production under a microscope. Osteoclast numbers per well were counted from histological images and plotted along the designated ROS inhibitor concentrations. Data are expressed as the mean ± SD of 3 independent experiments, each performed in triplicate, *p < 0.05 and **p < 0.01 (paired *t*-test).

Lee *et al*.^9^ identified a crucial role for ROS in RANKL-induced murine BMM differentiation into osteoclasts. Thus, we treated the mouse BMMs with various antioxidants such as superoxide dismutase (SOD), N-acetylcysteine (NAC), and diphenyleneiodonium (DPI) to see whether these ROS scavengers can prevent M-CSF- and RANKL-induced differentiation of osteoclast precursors into osteoclasts. SOD plays a crucial role in the superoxide detoxification process. NAC is a potent oxidant scavenger, and DPI is an inhibitor of flavoprotein, a Nox constituent. All the antioxidants used in this experiment inhibited osteoclast formation in a dose-dependent manner (Fig. 4). These parallel relationships of ROS levels with the potentials of BMMs to differentiate into osteoclasts support our view that intracellular ROS play important role for the differentiation of BMMs into osteoclasts.

### H_2_O_2_ rescued the osteoclast differentiation defect of *gp91^phox−/−^* BMMs

**Figure 5:**
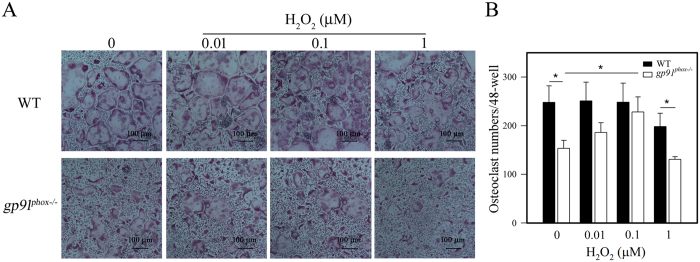
Differential effects of H_2_O_2_ on osteoclast differentiation in wild-type and *gp91*^*phox*−/−^ BMMs. BMMs were treated with H_2_O_2_ at the indicated concentrations osteoclast differentiation was induced. (**A**) Effects of H_2_O_2_ on osteoclast differentiation were visually observed under a microscope. (**B**) Numbers of osteoclasts per well were counted and plotted along the concentrations of H_2_O_2_. Data are expressed as the mean ± SD of 3 independent experiments, each performed in triplicate, *p < 0.05 and **p < 0.01 (paired *t*-test).

Membrane-bound gp91^phox^ is one component of the Nox2 complex that generates superoxide anions from oxygen. Superoxide spontaneously forms H_2_O_2_, which undergoes further reactions to generate ROS. Thus, we tested if H_2_O_2_ treatment could rescue the osteoclast differentiation defect of gp91^phox^ knockout BMMs. To accomplish this, *gp91*^*phox*−/−^ BMMs were subjected to RANKL-induced osteoclast differentiation in the presence or absence of H_2_O_2_, and we evaluated osteoclast differentiation. As shown in Fig. 5, 0.1 μM H_2_O_2_ completely rescued the defective osteoclast differentiation in *gp91*^*phox*−/−^ BMMs to levels seen in wild-type BMMs.

### Gp91^phox^-derived superoxide enhanced RANKL-induced NFATc1 expression in osteoclasts

**Figure 6:**
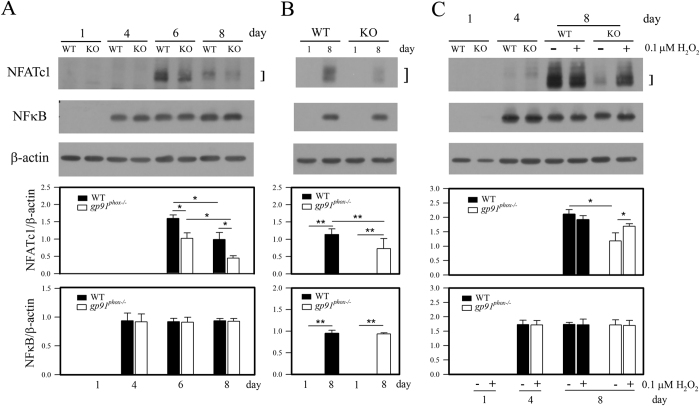
Effect of H_2_O_2_ on NFATc1 and NF-κB expression in osteoclasts from wild-type and *gp91*^*phox*−/−^ mice during RANKL-induced osteoclast differentiation. Data are expressed as the mean ± SD of 3 independent experiments, each performed in triplicate, *p < 0.05 and **p < 0.01 (paired *t*-test). (**A,B**) Differential NFATc1 expression between wild-type and *gp91*^*phox*−/−^ cells during osteoclast differentiation is shown by western blotting. (**C**) BMMs from wild-type and *gp91*^*phox*−/−^ mice were subjected to RANKL-induced osteoclast differentiation following treatment with or without 0.1 μM H_2_O_2_. After 4 (osteoclast precursor) and 8 (osteoclast) days, expression of NFATc1 and NF-κB was assessed by western blotting.

RANKL signaling activates multiple downstream signaling pathways required for osteoclast differentiation including NFATc1, NF-κB and AP-1. In particular, NFATc1 represents the master switch for osteoclast differentiation downstream of RANKL. Thus, we measured expression levels of NF-κB, AP-1, and NFATc1 in *gp91*^*phox*−/−^ and wild-type BMMs during RANKL-induced osteoclast differentiation. BMMs were incubated with M-CSF for 3 days to induce differentiation to osteoclast precursors. Osteoclast precursors were cultured with 30 ng/mL M-CSF and 50 ng/mL RANKL for an additional 4 days to promote differentiation into mature osteoclasts. We monitored NFATc1 and NF-κB protein levels by western blotting during this process. As expected, BMMs from wild-type mice expressed NFATc1 in response to RANKL, reaching a maximum level on day 6 and declining afterwards. In sharp contrast, BMMs from *gp91*^*phox*−/−^ mice showed blunted NFATc1 expression during RANKL stimulation (Fig. 6A,B). However, we did not notice a significant difference in NF-κB levels between wild-type and *gp91*^*phox*−/−^ BMMs upon RANKL stimulation (Fig. 6A,B). These results indicate that NF-κB expression is not affected by gp91^phox^ deficiency during RANKL-induced osteoclast differentiation. Although RANKL induces other osteoclast-specific genes including AP-1 and MAPKs during osteoclast differentiation^21^,^22^,^36^, we did not detect any noticeable effects of gp91^phox^ on the levels of AP-1 and phosphorylated MAPKs in both osteoclast precursors and osteoclasts regardless in the presence and absence of RANKL during differentiation culture (data not shown), suggesting that MAPKs and AP-1 may be regulated independently of gp91^phox^-derived superoxide signaling pathways.

We then asked whether gp91^phox^ cells defect in RANKL-induced NFATc1 expression could be rescued by H_2_O_2_ treatment. To address this, we monitored NFATc1 expression in differentiating osteoclasts in the presence or absence of 0.1 μM H_2_O_2_. Addition of H_2_O_2_ to *gp91*^*phox*−/−^ BMMs greatly raised NFATc1 expression levels upon RANKL stimulation to levels almost comparable to the wild-type BMMs on day 8 (Fig. 6C). This suggests that ROS play a critical role in inducing NFATc1 expression in response to RANKL stimulation. H_2_O_2_ treatment did not significantly affect wild-type BMMs.

NFATc1 represents the master switch for regulating osteoclast differentiation, functioning downstream of RANKL. Thus, our results indicate that gp91^phox^-derived superoxide induces NFATc1 expression in response to RANKL stimulation, implicating gp91^phox^ in promoting osteoclast differentiation downstream of RANKL. Although RANKL induces other osteoclast-specific genes including AP-1 and MAPKs during osteoclast differentiation^21^,^22^,^36^,^37^, we did not detect any noticeable effects of gp91^phox^ on the levels of AP-1 and phosphorylated MAPKs (data not shown), suggesting that MAPKs and AP-1 are regulated independently of gp91^phox^-derived superoxide signaling pathways.

## Discussion

Although ROS have been shown to act as a cellular secondary messengers, the involvement of ROS signaling pathways in RANKL-mediated osteoclast differentiation remain unclear. In this study, we demonstrated that *gp91*^*phox*−/−^ -derived superoxide participates in osteoclast differentiation from BMMs. These conclusions were verified by *in vivo* observations that the femurs from *gp91*^*phox*−/−^ mice had fewer osteoclasts and higher bone density than femurs from wild-type mice. Levels of osteoclast-specific markers and TRAP activity were assessed in osteoclasts differentiating in response to M-CSF/RANKL. Consistent with the *in vivo* results, the osteoclasts from *gp91*^*phox*−/−^ mice expressed significantly lower levels of osteoclast markers compared to osteoclasts from wild-type mice. We also noticed that upon RANKL stimulation, the BMMs from *gp91*^*phox*−/−^ mice could not efficiently upregulate NFATc1, the master switch for regulating osteoclast differentiation. Addition of H_2_O_2_ to the differentiation cultures rescued the differentiation defects of BMMs from *gp91*^*phox*−/−^, by raising osteoclast numbers and RANKL-induced NFATc1 expression almost to wild-type levels. Conversely, antioxidants or ROS scavengers hampered osteoclast differentiation. These data clearly suggest that *gp91*^*phox*−/−^ -derived superoxide contributes to osteoclast differentiation by enhancing NFATc1 expression, and serves as a secondary messenger downstream of RANKL.

Nox-derived ROS are crucial for RANKL-induced osteoclast differentiation. Several Nox isoforms, such as Nox1, Nox2 (gp91^phox^), and Nox4 are known to mediate osteoclastogenesis in BM macrophages and osteoclasts. However, it is not known which isoforms participate in the specific stages of osteoclast differentiation. Yang *et al*.^38^ were the first to identify Nox4 as an NADPH oxidase expressed in BM-derived osteoclasts. They reported that Nox4 expression was increased during the course of osteoclastogenesis. These authors also demonstrated that Nox4, but not Nox2, is involved in RANKL-induced ROS formation, showing that antisense Nox4 oligonucleotides reduced osteoclastic superoxide generation and resorption pit formation^30^,^38^. Consistent with these results, *Nox4*^−/−^ mice showed reduced osteoclast numbers and markers, with higher bone density^31^. In contrast, Sasaki *et al*. reported that Nox4 siRNA did not affect RANKL-dependent osteoclast differentiation^33^. Consistent with their results, Nox4 does not seem to be involved in acute TRAF6-mediated RANKL-induced signaling.

Nox4 is upregulated and becomes detectable in BMMs only after stimulation with RANKL/M-CSF, and thereby differentiation into osteoclasts^30^,^31^,^33^. Nox4 expression is a separate, later event during the course of differentiation^31^,^33^. In contrast to Nox4, Nox2 mRNA expression is highest in early stages of differentiation and reduces as RANKL-induced differentiation proceeds. As Nox2 expression decreases, reciprocal upregulation of Nox1 and Nox3 transcripts occurs^32^. In contrast to these findings, earlier reports indicated that Nox2 levels were higher in mature osteoclasts compared to precursors as determined through RT-PCR and immunocytochemistry^9^,^39^. These studies reported that Nox1, not Nox2, is the main producer of ROS during osteoclastogenesis. In general, previous reports on the roles of Nox isoforms in osteoclast differentiation are controversial. Unlike BM-derived osteoclast precursors, the mouse macrophage cell line, RAW 264.7 cells constitutively express abundant Nox2 mRNA at a level 1,000 times greater than Nox1 in response to RANKL, and Nox4 is not detectable^32^.

The reported discrepancies in Nox isoform expression levels during osteoclast differentiation may be mainly due to dynamic expression kinetics of each Nox isoform. Such conflicting findings may indicate that different Nox isoforms contribute to osteoclast differentiation at distinct timings, and therefore Nox isoforms may play non-overlapping or sequential roles for osteoclast formation depending on the differentiation stage. In fact, knockdown of any one Nox isoform often fails to cause noticeable changes in RANKL-mediated ROS production or osteoclast formation. For example, BMMs from Nox1 as well as Nox2 knockout mice generated ROS in response to RANKL and also differentiated into osteoclasts to the same degree as wild-type cells^33^. This results seemingly contrast our observations. Interestingly, Nox1 and Nox2 siRNAs significantly suppressed ROS generation and osteoclast formation in *Nox2*^−/−^ and *Nox1*^−/−^ cells, respectively. Therefore, there may be a flexible compensatory mechanism between Nox isoforms to facilitate osteoclast differentiation^32^. Thus, we are in favor of the view that Nox2 is involved in osteoclast differentiation from osteoclast precursor as a downstream mediator of RANKL, and is especially involved in NFATc1 induction. Therefore, Nox2 may play a unique role in differentiation by enhancing NFATc1-mediated transcriptional activity. Nakanishi *et al*.^3^ provided some evidence that Nox2 is essential for RANK expression in rat BMMs. Furthermore, mitochondrial redox signaling cross-talks with Nox complexes^16^. Considering that pre-osteoclast mitochondria produce ROS upon RANKL stimulation, it is possible that RANKL-mediated ROS formation is impaired in Nox2-deficient cells. Therefore, Nox2 may play a distinct role from other Nox homologs by providing mitochondrial ROS to BMMs during osteoclast differentiation. Supplementary H_2_O_2_ may substitute for Nox2 deficiency by enhancing RANKL-induced NFATc1 expression.

RANKL stimulation is coupled with NFATc1 activation, and a sustained NFATc1-dependent transcriptional program may represent the master switch for regulating osteoclast differentiation downstream of RANKL. In line with this, NFATc1-deficient embryonic stem cells fail to differentiate into osteoclasts in response to RANKL stimulation, and ectopic NFATc1 expression allows efficient osteoclast differentiation in the absence of RANKL signaling. We found that gp91^phox^ deficiency markedly attenuated RANKL-induced NFATc1 expression and led to impaired osteoclast differentiation. Based on these results, we conclude that gp91^phox^-derived superoxide participates in osteoclast differentiation by assuring a sustained NFATc1-dependent transcriptional program. Further studies are necessary to determine the precise mechanism by which gp91^phox^-derived superoxide cooperates with RANK signaling pathways to enhance NFATc1 activity.

## Materials and Methods

### Mice

Wild-type C57BL/6 J mice (Jackson Lab.) and those lacking the gp91phox gene^40^ were housed under specific pathogen-free conditions at the animal facility of Inha University. All procedures were conducted in accordance with institutional guidelines approved by the Animal Care and Use Committee of Inha University (INHA 131217-255). Mice used in these experiments were 6–8 - week - old males unless otherwise stated.

### Histologic and μCT analysis

For histological studies, bones were fixed in buffered formalin and subjected to μCT analysis (SkyScan, Brucker, Belgium), or decalcified in 10% EDTA, embedded in paraffin, sectioned, and stained for H&E and TRAP. Bone volume and density in a standard zone were determined using 6.5-μm voxel size at 80 kV and 80 μA. Bone samples were taken at 3000 msec per projection (500 total projections) and trabecular bone parameters (thickness, separation, and number) were analyzed by μCT^41^. Mouse body weight and bone weight were measured before experiments.

### Osteoclast formation

Bone marrow cells were isolated from 6–8-week-old male mice and cultured overnight in α-MEM (Hyclone) supplemented with 10% fetal bovine serum, 100 U/mL penicillin, 100 μg/mL streptomycin, and 10 ng/mL recombinant murine M-CSF (Peprotech). Subsequently, nonadherent cells were separated and adherent cells (2 × 10^4^ cells/96-well and 5 × 10^4^ cells/48-well) continuously cultured with 30 ng/mL M-CSF for 3 additional days to induce differentiation into osteoclast precursors. Osteoclast precursors were cultured for an additional 4 days with a combination of 30 ng/mL M-CSF and 50 ng/mL RANKL (Peprotech). Osteoclast formation was assessed by counting TRAP-positive cells with more than three nuclei. For ROS-related experiments, BMMs were treated with H_2_O_2_ or various antioxidants such as SOD, NAC, or DPI.

### TRAP staining

TRAP-positive cells were detected with a Leukocyte Acid Phosphatase Assay kit (Sigma) by following the manufacturer’s instructions. Briefly, cells were washed with PBS and fixed with a fixing solution containing citrate, acetone, and formaldehyde. Fixed cells were incubated with TRAP-staining solution for 1 h at 37 °C in the dark. After washing with water, cells were counterstained with hematoxylin. TRAP-positive cells containing three or more nuclei were counted under a light microscope.

### TRAP activity

Cells were incubated in 50 mM citrate buffer (pH 4.5) containing 10 mM sodium tartrate and 5 mM 4-nitrophenyl phosphate in 96-well plates for 30 min at 37 °C. The reaction mixtures were then transferred to a new 96-well plate containing an equal volume of 0.1 N NaOH. Absorbance was measured at 405 nm using Versamax microplate reader (Molecular Devices) equipped with SoftMax software (Molecular Devices).

### Quantitative real time PCR

Total RNA was extracted from cells using TRI reagent (MRC) according to the manufacturer’s instructions. Reverse transcription of total RNA was performed according to the protocol provided by Takara (Takara). PCR amplification of cDNA was carried out on an Applied Biosystems StepOne unit using SYBR Green PCR Master Mix and the following primers (forward and reverse, respectively): TRAP, 5′-ACG GCT ACT TGC GGT TTC A-3′ and 5′-TCC TTG GGA GGC TGG TCT T-3′; CTSK, 5′-GAA GAA GAC TCA CCA GAA GCA G-3′ and 5′-TCC AGG TTA TGG GCA GAG ATT-3′; GAPDH, 5′-CCT TCC GTC CTA CCC C-3′ and 5′-CCC AAG ATG CCC TTC ATG-3′. Amplification data were analyzed using the sequence detection software provided by Applied Biosystems.

### Measurement of NADPH oxidase activity

Superoxide production was measured by an isoluminol chemiluminescence assay^42^ in 96-well plates using a SpectraMax L microplate reader (Molecular Devices). Osteoclast precursors (1 × 10^5^) in PBSG (PBS with 0.9 mM CaCl_2_, 0.5 mM MgCl_2_, and 7.5 mM glucose) were added to each well of a 96-well plate with 125 μM isoluminol (Sigma) in 80 μL PBSG and 100 units HRP (Sigma) in 40 μL PBSG. After cells were incubated at 37 °C for 10 min, either 200 ng/mL PMA or 50 ng/mL RANKL was then injected into the wells by the automatic injector of the microplate reader. Chemiluminescence was detected as relative luminescence units by fast kinetic mode, and the amount of superoxide produced was determined using SoftMax PRO software. ROS levels in RANKL-treated osteoclasts were detected with 2′,7′-dichlorofluorescein diacetate (DCF-DA) as described previously^43^. DCF, a highly fluorescent compound formed from DCF-DA as a result of exposure to ROS, was detected by fluorescence spectroscopy with maximum excitation and emission spectra of 495 nm and 529 nm, respectively. The DCF-DA stained cells were also examined under a fluorescence microscope (Carl Zeiss, Oberkochen, Germany).

### Western blot analysis

Cell lysates were prepared as described previously^44^ and 20–30 μg of total protein was separated with SDS-PAGE. Resolved proteins were transferred onto polyvinylidene fluoride membranes (BioRad) which were probed with specific Abs against MAPKs (Cell Signaling), Akt (Cell Signaling), NF-κB (Santa Cruz), AP-1 (Santa Cruz), NFATc1 (Santa Cruz), or β-actin (Sigma). The membranes were incubated with the appropriate secondary antibody and then signals were developed using the ECL method (Amersham).

### Bone resorption assay

Cells were seeded on calcium phosphate plates (Corning osteo assay, Corning) and then cultured for 4 days with 30 ng/mL M-CSF and 50 ng/mL RANKL. Osteoclasts were removed using 0.2% Triton X-100 in 1 M NaCl to visualize resorption pits. Photographs of decalcified area were obtained under a light microscope at 40× magnification, and decalcified areas were measured using Image-Pro plus 4.5 software (MediaCybernetics).

### Statistical analysis

Two-tailed Student’s *t*-test (paired) or one-way ANOVA for Fig. 2E were performed using SigmaPlot11 software (Systat Software). Results were expressed as mean ± SD, and p values less than 0.05 were considered statistically significant.

## Additional Information

**How to cite this article**: Kang, I. S. and Kim, C. NADPH oxidase gp91^phox^ contributes to RANKL-induced osteoclast differentiation by upregulating NFATc1. *Sci. Rep.* **6**, 38014; doi: 10.1038/srep38014 (2016).

**Publisher's note:** Springer Nature remains neutral with regard to jurisdictional claims in published maps and institutional affiliations.
